# Transcriptome profiles of fatty acid metabolism-related genes and immune infiltrates identify hot tumors for immunotherapy in cutaneous melanoma

**DOI:** 10.3389/fgene.2022.860067

**Published:** 2022-09-19

**Authors:** Yunxian Dong, Zirui Zhao, Maijimi Simayi, Chufen Chen, Zhongye Xu, Dongming Lv, Bing Tang

**Affiliations:** ^1^ Department of Burn and Plastic Surgery, The First Affiliated Hospital of Sun Yat-sen University, Guangzhou, Guangdong, China; ^2^ Department of Gastrointestinal Surgery, The First Affiliated Hospital of Sun Yat-sen University, Guangzhou, Guangdong, China; ^3^ Department of General Surgery, The First People’s Hospital of Kashgar, Kashgar, China

**Keywords:** cutaneous melanoma, hot tumors, immunotherapy, precision medicine, fatty acid metabolism

## Abstract

**Background:** Immunotherapy with checkpoint inhibitors usually has a low response rate in some cutaneous melanoma (CM) cases due to its cold nature. Hence, identification of hot tumors is important to improve the immunotherapeutic efficacy and prognoses of CMs.

**Methods:** Fatty acid (FA) metabolism-related genes were extracted from the Gene Set Enrichment Analysis and used in the non-negative matrix factorization (NMF), copy number variation frequency, tumor mutation burden (TMB), and immune-related analyses, such as immunophenoscore (IPS). We generate a risk model and a nomogram for predicting patient prognoses and predicted the potential drugs for therapies using the Connectivity Map. Moreover, the NMF and the risk model were validated in a cohort of cases in the GSE65904 and GSE54467. At last, immunohistochemistry (IHC) was used for further validation.

**Results:** Based on the NMF of 11 FA metabolism-related DEGs, CM cases were stratified into two clusters. Cluster 2 cases had the characteristics of a hot tumor with higher immune infiltration levels, higher immune checkpoint (IC) molecules expression levels, higher TMB, and more sensitivity to immunotherapy and more potential immunotherapeutic drugs and were identified as hot tumors for immunotherapy. The risk model and nomogram displayed excellent predictor values. In addition, there were more small potential molecule drugs for therapies of CM patients, such as ambroxol. In immunohistochemistry (IHC), we could find that expression of *PLA2G2D*, *ACOXL*, and *KMO* was upregulated in CM tissues, while the expression of *IL4I1*, *BBOX1*, and *CIDEA* was reversed or not detected.

**Conclusion:** The transcriptome profiles of FA metabolism-related genes were effective for distinguishing CM into hot–cold tumors. Our findings may be valuable for development of effective immunotherapy for CM patients and for proposing new therapy strategies.

## 1 Introduction

Cutaneous melanoma (CM) is the most malignant of all skin tumors (Bray et al., 2018). Most of the patients with CM are diagnosed already at clinically detectable stage III with or without in-transit metastases, which is called high-risk resectable melanoma, and suffer from a high-risk relapse (up to 70%) when treated with surgery alone (Gershenwald et al., 2017; Schadendorf et al., 2018). Conventional surgical procedures and chemotherapies are difficult to effectively cure CM due to chemotherapy failure and severe adverse effects (Amaria et al., 2019). As a result, patients diagnosed at an advanced stage of CM have an extremely poor prognosis, with a 5-year survival rate of less than 10% (Schadendorf et al., 2018; Allais et al., 2021). Over the past decade, immunotherapy, such as immune checkpoint inhibitors (ICIs), has significantly prolonged CM patients’ overall survival (Hamid et al., 2019; Eggermont et al., 2021). Moreover, those ICIs usually have lower toxicity, high life quality, and treatment compliance in most CM patients. Unfortunately, the therapeutic efficacy and response rate in CM patients remain low. On the other hand, not all CM patients benefit from ICIs, although immunotherapy had achieved a lot. For example, programmed cell death protein 1(PD-1) inhibitor therapy usually has a response rate of one-third in CM (Galon and Bruni, 2019; Salmon et al., 2019; Tang et al., 2020).

The effectiveness of immunotherapy depends on the levels of circulating immune components in the body, CD8^+^ T cell infiltration, and proliferative ability in the tumors (Galon and Bruni, 2019; Salmon et al., 2019; Lopez de Rodas and Schalper, 2021). According to the immune infiltration level of tumors, we could classify tumors into two categories, “hot” and “cold”. The terms “hot” and “cold” are used to refer to T cell-infiltrated, inflamed but non-infiltrated, and non-inflamed tumors (Galon and Bruni, 2019). The hot tumors with high immune infiltration, particularly for CD8^+^ T cells, usually are sensitive to immunotherapy because the pre-stored immune cells can effectively attack tumor cells following immunotherapy, such as checkpoint inhibitors (Ochoa de Olza et al., 2020; Too et al., 2021). Therefore, it is necessary to apply different treatment strategies for cold and hot tumors. Actually, early transformation of cold tumors into hot tumors can improve the efficacy of immunotherapy and prognosis of patients (Ji et al., 2021; Li et al., 2021).

It is well known that because of hypoxia and aggressive growth, the TME usually has high oxidation of fatty acid (FA) metabolism. In CM, lipid metabolism is associated with the resistance to targeted therapeutic drugs by altered expression of the FA transporter FATP2 (Alicea et al., 2020). A metabolic reprogramming to FA oxidation (FAO) can regulate the adaptation of BRAF-mutated melanoma to MAPK inhibitors (Aloia et al., 2019). A strong FAO in dendritic cells can attenuate therapeutic responses to anti-PD-1 treatment in melanoma by modulating Wnt5a-β-catenin-PPAR-γ signaling (Zhao et al., 2018). Moreover, the fatty acid receptor GPR120 may be a new marker for human melanoma (Oh et al., 2010; Kleemann et al., 2018).

However, the relationship between FA metabolism-related genes and immunotherapy remains largely unclear in CM. Moreover, there is little information on reliable biomarkers to distinguish cold from hot tumors, including CM (Maleki Vareki, 2018; Long et al., 2022). In this study, we analyzed the FA metabolism-related genes and immune infiltrates in CM and after determining their prognostic values, we generated and validated a risk model and nomogram. In addition, we screened some small-molecule drugs. Our findings indicated the risk model and clusters were valuable for prognosis and predicting immunotherapeutic responses in CM patients.

## 2 Materials and methods

### 2.1 Date preparation

The RNA-seq profiles of 471 CM and one non-tumor samples of the cancer genome atlas (TCGA),as well as 555 non-tumor skin specimens of the Genotype-Tissue Expression Project (GTEx), were obtained from the University of California Santa Cruz (UCSC). Batch normalization was performed in the data set by the sva R package. The differentially expressed genes (DEGs) were analyzed using Counts format profiles and converted into TPM format profiles for further analyses using the limma R package (Stupnikov et al., 2021). Their clinical data, such as overall survival (OS), and copy number, were also downloaded from UCSC. Furthermore, RNA-seq profiles, survival status, and OS time of CM patients were obtained from the GSE65904 and GSE54467 datasets for external validation from Gene Expression Omnibus (GEO). To reduce statistical bias, CM samples with missing OS values or short OS values (< 30 days) were excluded from all cohorts. As a result, 447 patients in the TCGA cohort and 278 in the external validation cohort were used for analyses. In addition, the fatty acid (FA) metabolism-related gene sets, including M14568, M22474, M34207, M34208, M29237, M23047, M15179, M16838, M25445, M23048, M13605, M11966, M15385, M16969, M14401, M34091, M13290, M11936, M14177, M12558, M16551, M13480, M15568, M18199, M26370, M26153, M40674, M18978, M26866, M26251, M5935, M36310, M37191, M11673, M29570, M27727, M27719, M14690, M22174, M6999, M13591, M10250, M15813, M25014, M16181, M12334, M17829, M25044, M15938, M23782, M22457, M40405, M40498, M699, M6995, M9927, M15531, M27854, M39440, and M39596, containing 745 fatty acid metabolism-related genes were extracted. Their intersection genes were identified in the TCGA, GTEx, and external validation cohorts using the VennDiagram R package. Moreover, the tumor mutation burden data of these cohorts were obtained from the TCGA database.

### 2.2 FA DEGs and NMF

FA metabolism-related DEGs between the CM and non-tumor samples were identified, based on the criteria of a false discovery rate (FDR) < 0.05 and an absolute value Log2 fold change >1, using the limma R package in the TCGA and GTEx synthetic Counts matrix (Deng et al., 2021). This synthetic matrix had been normalized for identifying DEGs. Subsequently, 11 FA metabolism-related DEGs were identified using univariate Cox proportional hazard regression, and they had significant prognostic values (all *p* < 0.05) using the limma and survival R packages in the TCGA cohort (Zheng et al., 2021). These 11 FA metabolism-related DEGs with potent prognostic values were subjected to non-negative matrix factorization (NMF) analyses for sample clustering by NMF and survival R packages (Zhuo et al., 2020). The Kaplan–Meier survival curves of OS, t-distributed stochastic neighbor embedding (t-SNE), and Principal Component Analyses (PCA) of the clusters were analyzed using the survminer, Rtsne, ggplot2, and scatterplot3d R packages. Similar analyses were performed in the external validation cohort.

### 2.3 Copy number alterations

The copy number variation (CNV) frequencies of these FA metabolism-related prognostic genes were calculated, and their locations in human chromosomes were identified using the RCircos R package. For each prognostic gene, we compared the immune infiltration levels of CM patients with different somatic copy number alterations, such as deep deletion, arm-level deletion, and diploid/normal, using the Tumor Immune Estimation Resource (TIMER) (Kang et al., 2020).

### 2.4 Functional analysis

The potential functions and pathways of each set of genes in the cluster were analyzed using the Gene Set Enrichment Analyses (GSEA) software (https://www.gsea-msigdb.org/gsea/login jsp) and Curated gene set (kegg. v7. 4. symbols. gmt). Multi-GSEA diagrams were drawn using the plyr, grid, and gridExtra R packages.

### 2.5 Evaluation of immune infiltration

The immune infiltration of individual patients was analyzed using the CIBERSORT (R scrip v 1.03), and their immune scores, stromal scores, ESTIMATE (microenvironment) scores, and tumor purity were compared using the estimate R package, followed by visualizing them as the pheatmap using the ggtext packages (Gui et al., 2021). Subsequently, the immune cell infiltration, immune functions, and expression of genes for checkpoints were compared using the GSVA, GSEABase, ggpubr, reshape2, and limma R packages.

### 2.6 Tumor mutation burden analysis

The tumor mutation burden (TMB) data in the “Masked Somatic Mutation” type were processed by VarScan2 and analyzed in clusters using the maftools package (Koboldt et al., 2012). Furthermore, their overall survival was estimated by K–M survival analysis, and a box plot was made using the survminer, survival, ggplot2, ggpubr, and ggExtra R packages.

### 2.7 Immunotherapy-related exploration

According to the results of comprehensive immunogenomic analyses in The Cancer Immunome Atlas (TCIA), the response of each patient to immunotherapy was predicted and compared using the ggpubr R package (Charoentong et al., 2017). Additionally, the potential immunotherapeutic function of some drugs and their half-maximal inhibitory concentration (IC_50_) in CM patients were predicted based on the data from Genomics of Drug Sensitivity in Cancer (GDSC) using the pRRophetic R package (Gui et al., 2021).

### 2.8 Construction and assessment of the risk model and nomogram

CM patients were randomly divided into training and testing sets. A risk model was generated using the least absolute shrinkage and selection operator (LASSO) in the glmnet R package. The risk score formula (Zheng et al., 2021):

$$Riskscore=\overset{n}{\underset{k=1}{\sum }}{coef\;(gene\;}^{k})\;*\;expr\;{(gene\;}^{k})$$

The coef (gene) meant the coefficient of the gene in the risk model, and expr (gene) was the expression of the gene in the risk model. The status, survival time, heatmaps, Kaplan–Meier survival analyses, and receiver operating characteristic (ROC) curves of individual patients in the training, testing, and entire sets were also analyzed using the pheatmap, survival, and timeROC R packages. For validation, a risk score of each patient in the external validation cohort was calculated, and their survival was estimated by the Kaplan–Meier survival analysis. The specificity and sensitivity of this risk model were evaluated by ROC analysis.

According to patient's clinical data and risk scores, the independent prognostic factors for worse survival were identified using univariate Cox and multivariate Cox regression analyses and used for the generation of a nomogram using the nomogramEx and nomogramFormula R packages. The concordance of the nomogram was analyzed by the ROC curves using the timeROC and rms R packages.

### 2.9 Connectivity Map

For predicting potential small-molecule drugs that might reverse high risk in CM, the whole overlapping genes, including the upregulated and downregulated genes, were submitted into the CMap database (https://portals.broadinstitute.org/cmap/). The drugs with enrichment scores between −1 and 0 were considered candidate drugs for CM (all *p* < 0.05) (Gui et al., 2021).

### 2.10 Validation of protein expressions of FA metabolism-related genes by the Human Protein Atlas

#### 2.10.1 Database

The protein expression of the FA metabolism-related genes between CM and normal tissues was determined using immunohistochemistry (IHC) from the Human Protein Atlas database (HPA) as well as our own preserved patients’ tissue paraffin slides (Thul and Lindskog, 2018).

### 2.11 Histopathology

The tumor tissues were fixed in 10% formalin overnight and paraffin-embedded. The tissue sections (5 µm) were regularly stained with immunohistochemistry (IHC) staining. The stained tissue sections were photo-imaged and observed under a light microscope. The primary antibodies were used at 1:200 for *CIDEA* (Abcam, ab191193), *ACOXL* (Proteintech, 23366-1-AP), *PLA2G2D* (Abcam, ab47118), and 1:400 for *KMO* (Abcam, ab233529).

### 2.12 Statistical analysis

We made analyses with R version 3.6.3 (http://www.R-project.org) and its appropriate packages. All involved packages were described in MATERIALS AND METHODS. Data were analyzed with standard statistical tests as appropriate, while multiple testing was adjusted by the FDR method by R (Gui et al., 2021). Statistical significance was observed when *p* < 0.05.

## 3 Result

### 3.1 FA metabolism-related differentially expressed genes and NMF clusters

**FIGURE 1:**
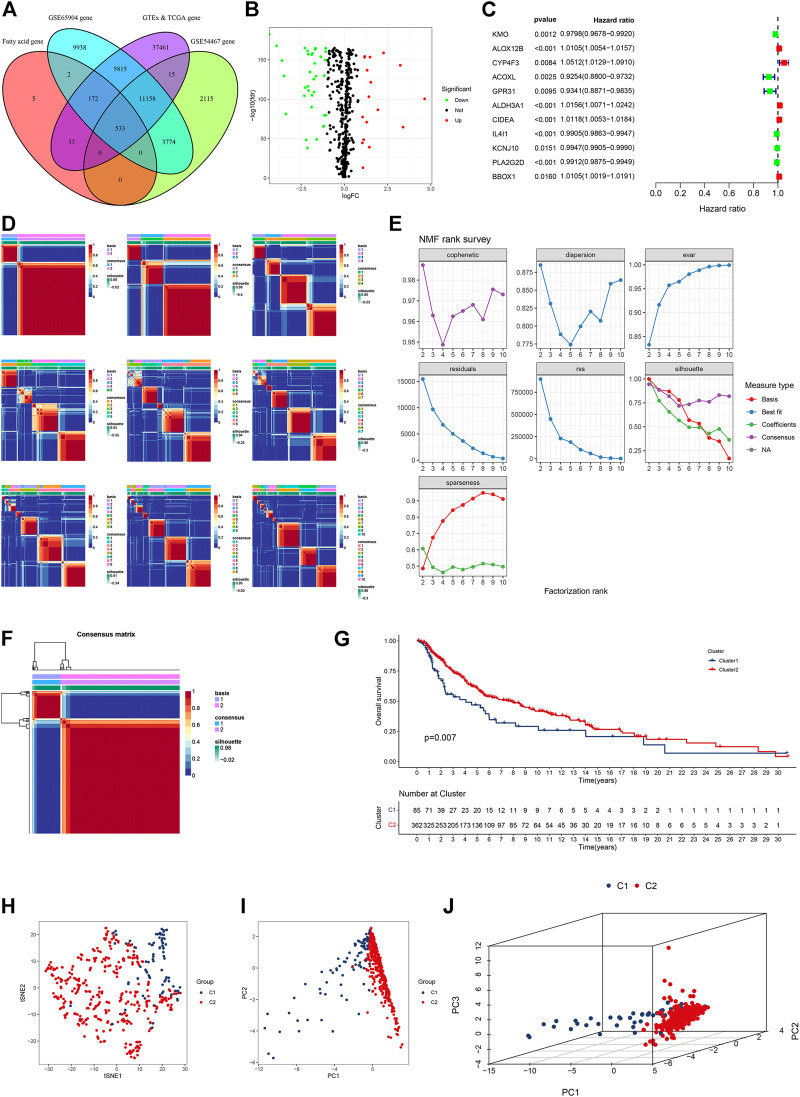
Identification of fatty acid metabolism-related genes in four gene subsets and clusters in the TCGA CM cohort. **(A)** Venn diagram displayed FA metabolism-related genes in CM cases from TCGA, GTEx, GSE65904, and GSE54467. **(B)** Volcano plot of 51 FA metabolism-related DEGs. **(C)** Forest plot of prognostic FA metabolism-related DEGs. **(D)** All the heatmaps of NMF consensus clustering. **(E)** NMF rank survey of cophenetic, dispersion, evar, residuals, rss, silhouette, and sparseness coefficients. **(F)** Heatmap of two clusters of CM. **(G)** Kaplan–Meier survival curves of OS in these clusters of CM cases. **(H–J)** t-SNE, PCA, and 3D PCA separated two clusters of CM. A *p* value of <0.05 was considered to indicate a statistically significant difference.

A total of 745 FA metabolism-related genes were identified in CM from the GSEA (Supplementary Table S1). Similarly, 533 of them were identified in CM from the GTEx, TCGA, GSE65904, and GSE54467 (Figure 1A). There were 51 DEGs in the TCGA cohort. Of them, 17 were upregulated and the others were downregulated (Figure 1B). Further analyses indicated that 11 DEGs were associated with the prognosis of CM (all *p* < 0.05, Figure 1C). Of them, *ALOX12B*, *CYP4F3*, *ALDH3A1*, *CIDEA*, and *BBOX1* were upregulated in CM, while the others were downregulated.

Based on comprehensive correlation coefficients and all heatmaps, we found the optimal total cluster number was set to k = 2 (Figures 1D,E). The heatmap (k = 2) indicated a clear boundary (Figure 1F). Compared with cluster 2, patients in cluster 1 had a worse OS (Figure 1G). To further verify the cluster distribution, the t-SNE, 2D PCA, and 3D PCA analyses clearly separated these prognostic genes (Figures 1H–J).

### 3.2 External verification of NMF clusters and analyses of copy number in CM

**FIGURE 2:**
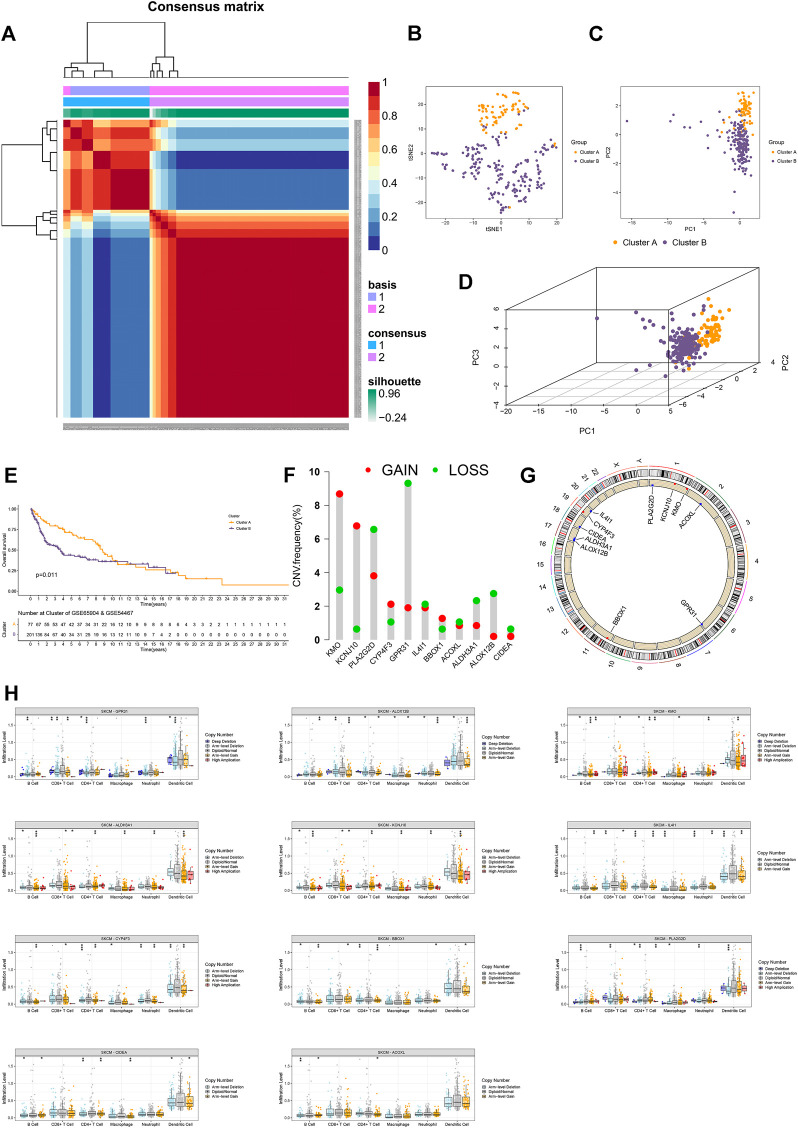
External validation and copy number analyses. **(A)** Heatmap of two clusters of cases in the external validation cohort. **(B–D)** t-SNE, PCA, and 3D PCA separated two clusters of CM cases in the external validation cohort. **(E)** Kaplan–Meier survival curves of OS in two clusters of CM cases in the external validation cohort. **(F)** CNV frequency of 11 FA metabolism-related genes. **(G)** CNV of prognostic genes on RCircos 2D track plot with the human genome. **(H)** Different types of immune infiltrates among samples with copy number of the indicated genes. Ns means no significant difference, *p < 0.05, **p < 0.01 and ***p < 0.001. A *p* value of <0.05 was considered to indicate a statistically significant difference.

In the external verification cohort, the optimal total cluster number was k = 2 in NMF. The difference between the two clusters and their NMF ranks are displayed (Figure 2A, Supplementary Figures S1A,B). In addition, the t-SNE, PCA, and Kaplan–Meier survival curves of OS analyses revealed their distribution, and patients in cluster B had a worse OS in this population (Figures 2B–E).

Next, we calculated the CNV frequency of 11 prognostic genes and located them in human chromosomes. The percentages of gain CNV of *KMO, KCNJ10, CYP4F3,* and *BBOX1* were higher than those of the loss, while the frequency of gain CNV of *PLA2G2D, GPR31, IL4I1, ACOXL, ALDH3A1, ALOX12B,* and *CIDE*A was lower than that of loss (Figure 2F). As a result, the *KMO, KCNJ10, CYP4F3,* and *BBOX1* genes were marked in red, and the *PLA2G2D, GPR31, IL4I1, ACOXL, ALDH3A1, ALOX12B,* and *CIDEA* were marked in blue on RCircos 2D track plots (Figure 2G). Furthermore, we explored the changes in immune cell infiltration with a copy number alteration of prognostic genes in CM. Alterations of these genes were associated with infiltration levels of CD8^+^ T cells or other immune cells (Figure 2H). These indicated that clusters of CM with varying prognostic genes had different immune microenvironments, leading to different responses to immunotherapy (O'Sullivan et al., 2019).

### 3.3 Analyses of the immune microenvironment and TMB in CM

**FIGURE 3:**
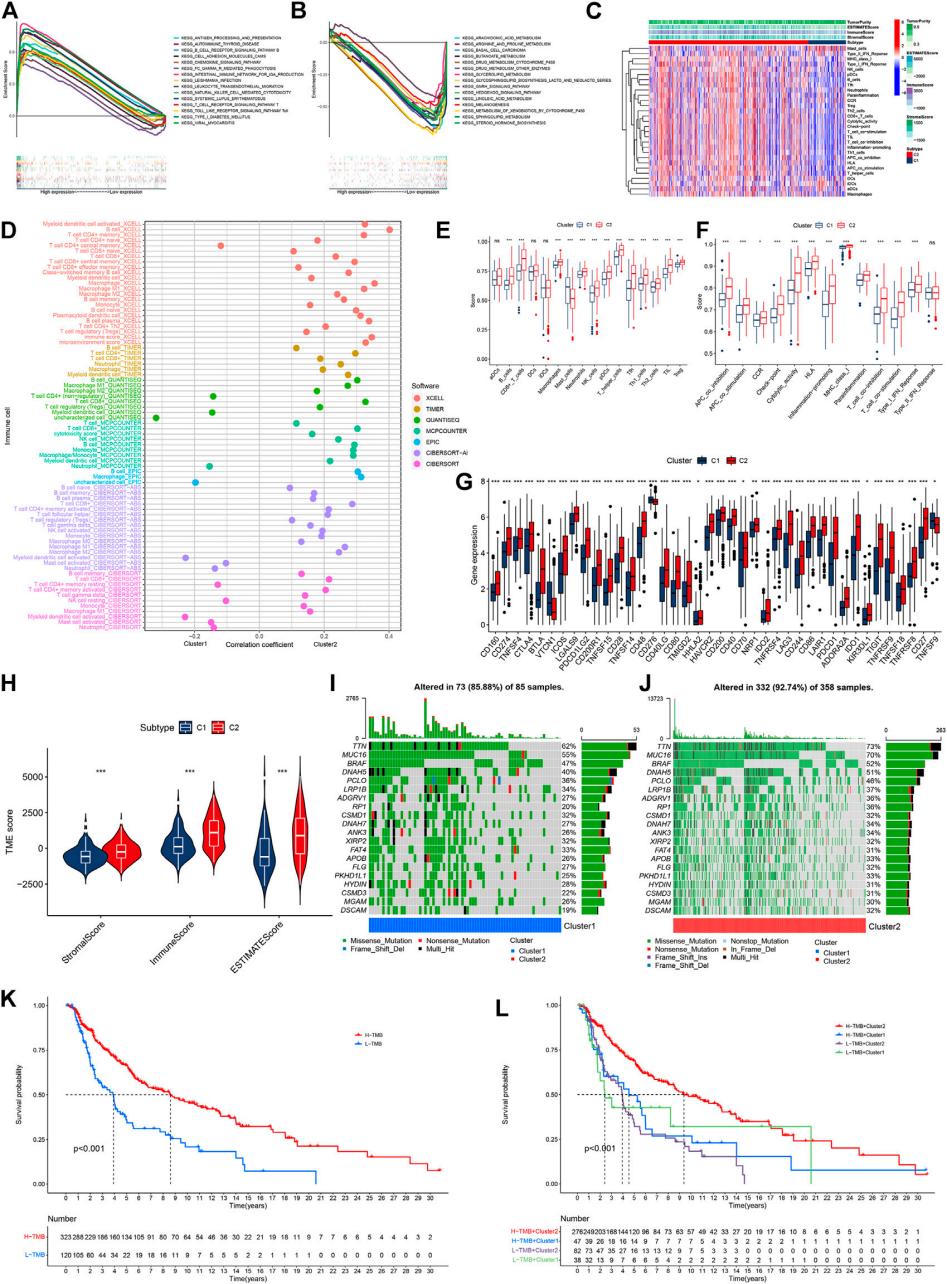
Analyses of tumor immune characteristics and TMB in these two clusters of CM cases. **(A)** Multi-GSEA analyses of cluster 2 CM cases. **(B)** multi-GSEA analyses of cluster 1 CM cases. **(C)** Heatmap of immune infiltrates between two clusters of CM cases. **(D)** Correlation coefficient of immune infiltrates in two clusters of CM cases. **(E,F)** Single-sample GSEA scores of immune cells and immune functions in two clusters of CM cases. **(G)** Comparisons of genes for expression of 40 checkpoints between two clusters of CM cases. **(H)** Comparisons of immune-related scores in two clusters of CM cases. **(I,J)** Waterfall plot of TMB of individual patients in cluster 1 and cluster 2. **(K)** Kaplan–Meier analysis of OS between the low- and high-TMB groups of CM cases. **(L)** Kaplan–Meier analysis of OS among four groups of CM cases. *p < 0.05, **p < 0.01, and ***p < 0.001. A *p* value of <0.05 was considered to indicate a statistically significant difference.

We performed GSEA to explore the biological functions of these clusters. Cluster 2 and cluster 1 of genes were involved in the top 15 pathways (all *p* < 0.05, FDR <0.05, |NES|>1.8, Supplementary Figures S1C,D). In cluster 2, almost all of the enriched pathways were associated with immunity, such as chemokine signaling, and natural killer cell-mediated cytotoxicity (Figure 3A) (Chow and Luster, 2014; Taniguchi and Karin, 2018). On the contrary, six out of the top 15 pathways enriched by the genes in cluster 1 were associated with FA metabolism, such as arachidonic acid metabolism, four pathways were related to tumor growth, and two were related to drug metabolism (Figure 3B). According to the heatmap of immune cell infiltration, we felt that the cluster 2 had a higher immune cell infiltration level, lower tumor purity, and more active immunity (Figure 3C). In the immune cell bubble chart, cluster 2 of CM had more immune cell infiltrates (Figure 3D, Supplementary Data Sheet S1). Based on the single-sample GSEA scores for immune cells and immune functions, 13 types of immune cells, such as CD8^+^ T cells, and 12 immune functions, such as inflammation-promoting and check-point, had a higher score in cluster 2 of CM (ns means no significant difference, *p < 0.05, **p < 0.01, and ***p < 0.001, Figures 3E,F). Of course, almost all immune checkpoint genes, such as *CD274 (PD-L1), CTLA4, HAVCR2 (TIME3),*and *LAG3*, displayed a higher activation in cluster 2 (Figure 3G) (Galon and Bruni, 2019). Cluster 2 had a high immune score, stromal score, and ESTIMATE score (microenvironment score, Figure 3H). Therefore, cluster 2 of CM was considered the hot tumor to respond to immunotherapy because cluster 2 of CM displayed the characteristics of hot immune tumors, such as a high degree of CD8^+^ T cells, high immune score, more active immune functions, and higher expression of *CTLA4*, *TIM3,* and *LAG3* (Galon and Bruni, 2019; Zheng et al., 2021). Previous studies have shown that a high TMB is significantly associated with improved OS and benefits from immunotherapy, such as *CTLA-4* blockade, and the TMB has been considered a potential immunotherapy parameter (Snyder et al., 2014; Mahoney et al., 2015; Chan et al., 2019). Hence, we analyzed the TMB of CM in the TCGA cohort to explore the responses of these clusters of CM to immunotherapy. The TMB frequency in cluster 2 of CM (92.74%) was higher than that of cluster 1 (85.88%) (Figures 3I,J). Consistently, the high-TMB group (H-TMB) of CM patients had better OS than those in the low-TMB group (L-TMB) (Figure 3K). Stratification analysis revealed that CM patients with high TMB displayed a better OS than those with low-TMB in both clusters (Figure 3L).

### 3.4 Investigation in immunotherapy

**FIGURE 4:**
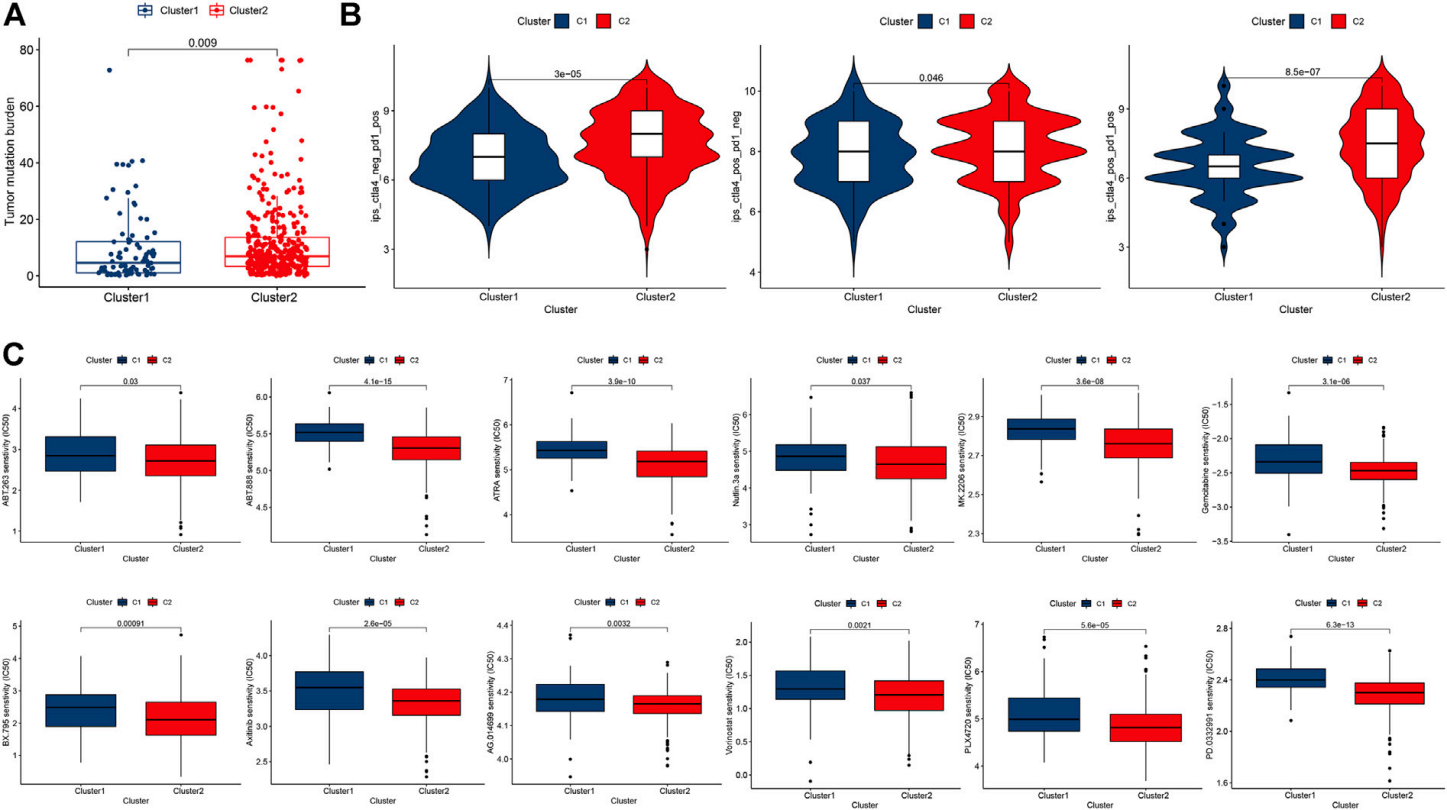
Comparisons of TMB, immune checkpoint gene expression, and drug sensitivity between two clusters of CM cases. **(A)** Levels of TMB in two clusters of CM cases. **(B)** *PD-1* and/or *CTLA4* in two clusters of CM cases. **(C)** Prediction of potential therapeutic drugs IC_50_ in two clusters of cases. A *p* value of <0.05 was considered to indicate a statistically significant difference.

Compared with cluster 1, CM in cluster 2 possessed significantly higher TMB (Figure 4A). Accordingly, we investigated immune checkpoint-related scores in these clusters of CM. Compared with cluster 1 CM, cluster 2 CMs had significantly higher both *PD-1* and *CTLA4* immunophenoscore (IPS, Figure 4B). In addition, CM patients in cluster 2 were likely to be more sensitive to 12 potential immunotherapy-related drugs with lower IC_50_ than those in cluster 1 (Figure 4C) (Haikala et al., 2019; Song et al., 2019).

### 3.5 Risk model and external verification

**FIGURE 5:**
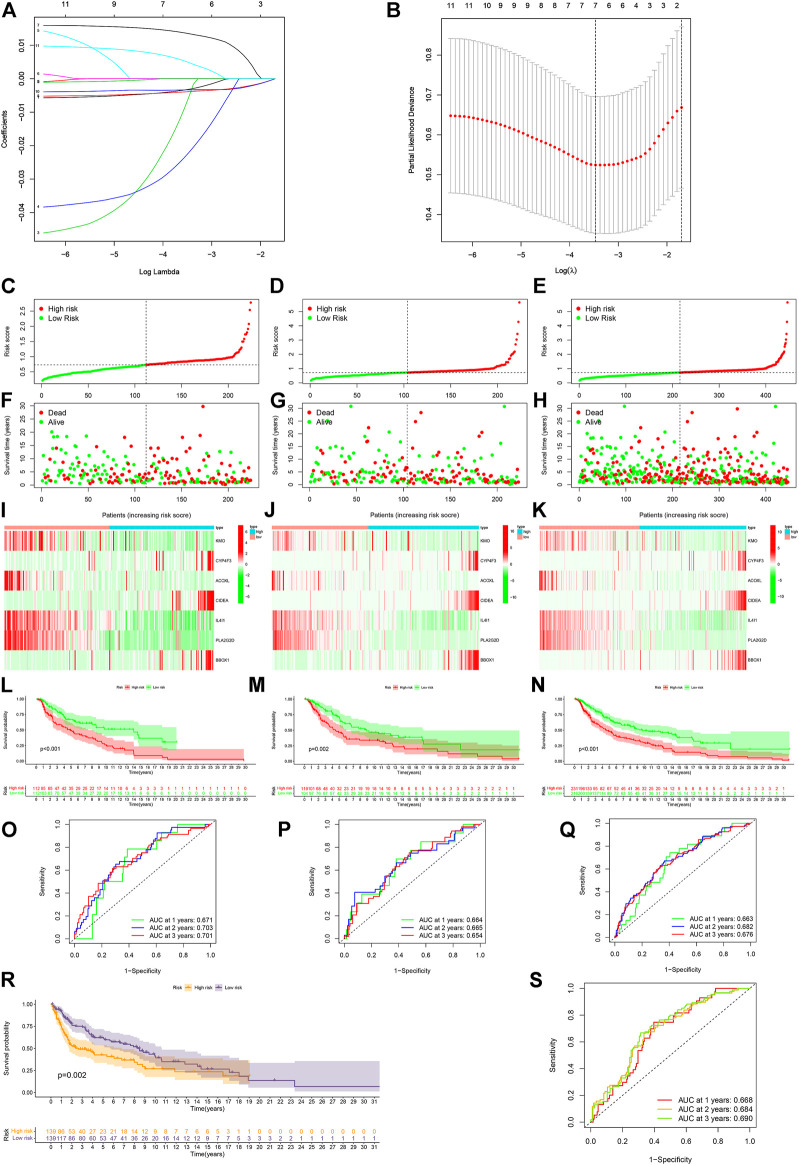
Construction and validation of the risk model. **(A,B)** Constructing a risk model of seven genes by LASSO regression. **(C–E)** Risk scores of the training, testing, and entire sets, respectively. **(F–H)** Survival status of individual cases between the low- and high-risk groups in the training, testing, and entire sets. **(I–K)** Heatmap of seven gene expression in the training, testing, and entire sets of CM cases. **(L–N)** Kaplan–Meier analysis of OS between the low- and high-risk groups of CM cases in the training, testing, and entire sets. **(O–Q)** ROC curves for 1-, 2- and 3-year OS of CM cases in the training, testing, and entire sets. **(R)** External validation of the risk model. **(S)** ROC curves for 1-, 2- and 3-year OS of CM case in the external validation cohort. A *p* value of <0.05 was considered to indicate a statistically significant difference.

With the LASSO regression analyses, a risk model of seven genes was established after control of the first-rank value of Log(λ) at the minimum likelihood of deviance (Figures 5A,B). The risk score formula was Risk score = KMO × (−0.0032) + CYP4F3 × (−0.0054) + ACOXL × (−0.0220) + CIDEA × 0.0135 + IL4I1 × (−0.0039) + PLA2G2D × (−0.0033) + BBOX1 × 0.0048. In addition, analyses of the survival status, survival time, expression of seven genes, and OS of patients clearly separated between low- and high-risk groups of CM patients in the training, testing, and entire sets of cases (Figures 5C–N). Hence, CM patients in the low-risk group displayed a better prognosis. The AUC for 1-, 2- and 3-year OS in the training set of cases was 0.671, 0.703, and 0.701, that of the testing set of cases was 0.664, 0.665, and 0.654, and that of the entire sets of cases was 0.663, 0.682, 0.676, respectively (Figures 5O–Q). After analyzing the clinical characteristic of the patients, we found our risk model was also available in age, gender, tumor stage, T stage. and N stage (Supplementary Figure S2A). Furthermore, stratification of CM patients was carried out into low- and high-risk groups, and the t-SNE, 2D PCA, and 3D PCA clearly separated them (Supplementary Figures S3A). Further validation revealed that CM patients in the low-risk group exhibited significantly better OS than those in the high-risk group (*p* = 0.002, Figure 5R). The AUC for 1-, 2-, and 3-year OS in the external validation cohort were 0.668, 0.684, and 0.690, respectively (Figure 5S).

### 3.6 Construction and assessment of a nomogram

**FIGURE 6:**
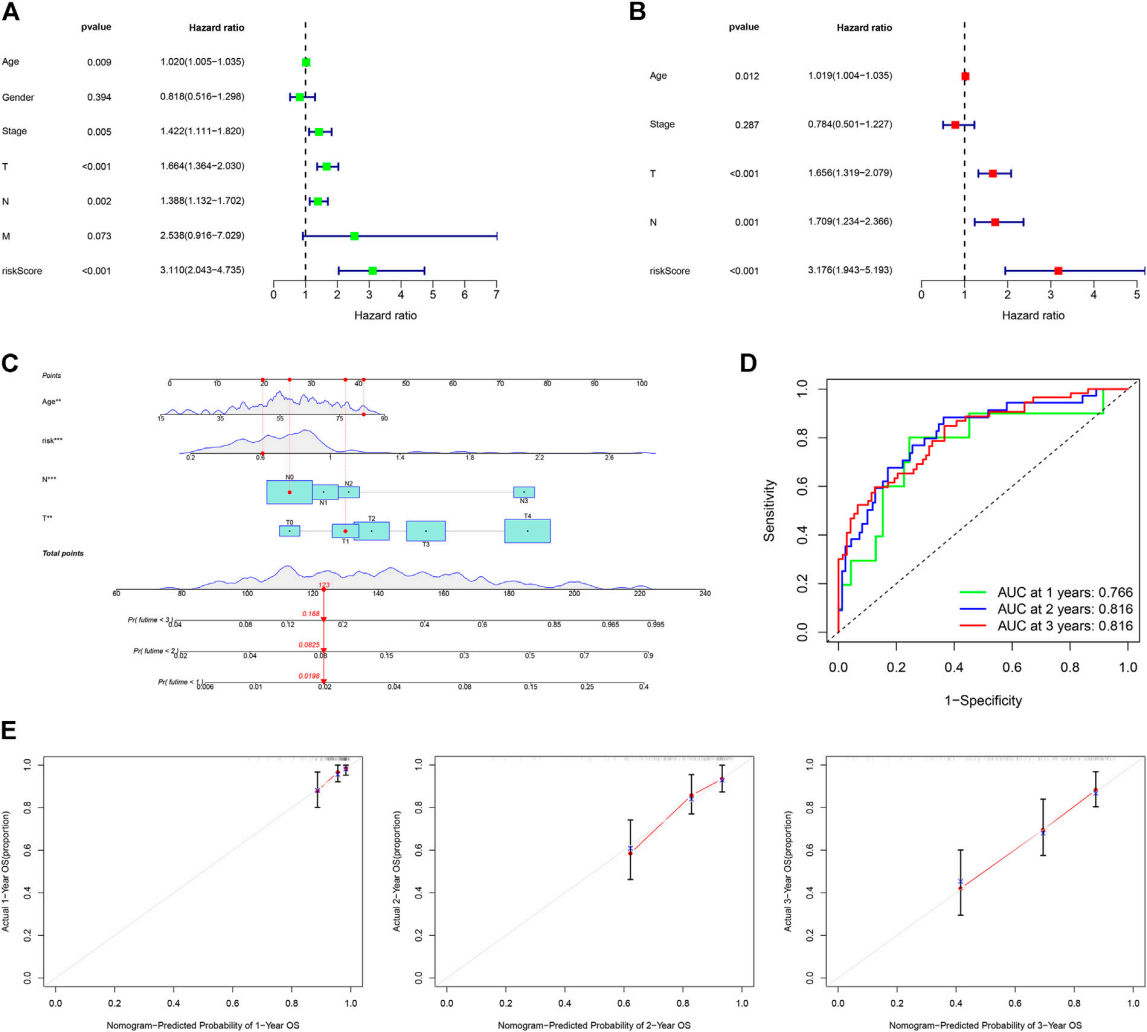
Construction and assessment of a nomogram. **(A,B)** Univariate and multivariate Cox analyses of clinical characteristics to identify independent risk factors for worse OS. **(C)** A nomogram based on age, T, N stage, and risk scores. **(D)** ROC curves for 1-, 2- and 3-year OS of CM cases using the nomogram. **(E)** Calibration ROS curves for 1-, 2- and 3-year OS.

The univariate Cox (uni-Cox) and multivariate Cox (multi-Cox) regression analyses indicated that patient’s age, tumor T stage, tumor N stage, and risk score were independent risk factors for prognosis of CM patients. Their independent risk factors were associated with a worse prognosis for CM patients with hazard ratios and a 95% confidence interval (CI) (Figures 6A,B). With these four independent prognostic indexes, a nomogram was generated (Figure 6C). The AUC of a nomogram for 1-, 2-, and 3-year OS of CM patients was 0.766, 0.816, and 0.816, respectively (Figure 6D). The calibration plots of the nomogram for 1-, 2-, and 3-year OS of CM patients had a good concordance with the prediction (Figure 6E).

### 3.7 The potential value of the risk model in clinical application

**FIGURE 7:**
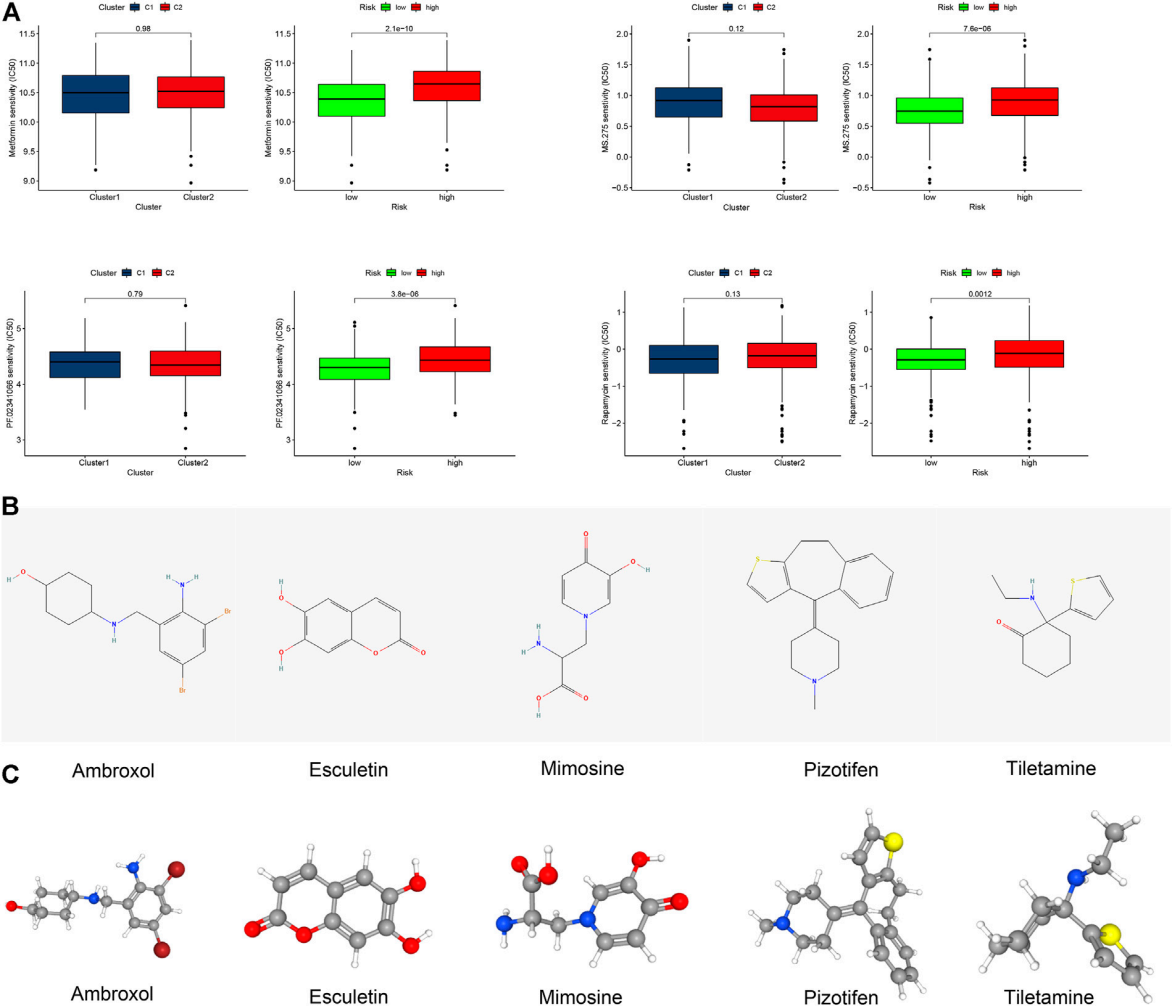
Clinical prediction of drug sensitivity in CM cases between two clusters or two risk groups. **(A)** Four potential drugs solely had significant IC_50_ differences between these two risk groups. **(B,C)** 2D structure illustrations and the 3D structure tomography of five candidate small-molecule drugs for CM. A *p* value of <0.05 was considered to indicate a statistically significant difference.

Among the top 15 signaling pathways, 11 signaling pathways were associated with immunity, such as the T cell receptor signaling (all *p* < 0.05, FDR <0.05, |NES|>2.3) (Supplementary Figure S3B) (Courtney et al., 2018). The immune heatmap exhibited that CM in the low-risk group had more types of immune cells (Supplementary Figure S3C, Supplementary Data Sheet S2). The levels of CD8^+^ T, B cell infiltrates, and other types of immune cells were also correlated with lower risk scores (Supplementary Figures S3D). Furthermore, CM in the low-risk group had higher immune scores (Supplementary Figures S3E). Thus, we tried to explore potential immunotherapy-related drugs for these risk groups of CM. As a result, we found that four drugs, such as metformin, had significantly different IC_50_ between both risk groups of CM (Figure 7A) (O'Sullivan et al., 2019; Cha et al., 2018). A total of five most related small-molecule drugs, ambroxol, tiletamine, mimosine, esculetin, and pizotifen, were identified (all *p* < 0.05), based on upregulated and downregulated gene expression between the low- and high-risk groups of CM (Supplementary Data Sheet S3) (Gui et al., 2021). Their 2D and 3D structure tomography are is in Figures 7B,C.

### 3.8 Verification of the protein expression of FA metabolism-related genes in normal skin and CM tissue

**FIGURE 8:**
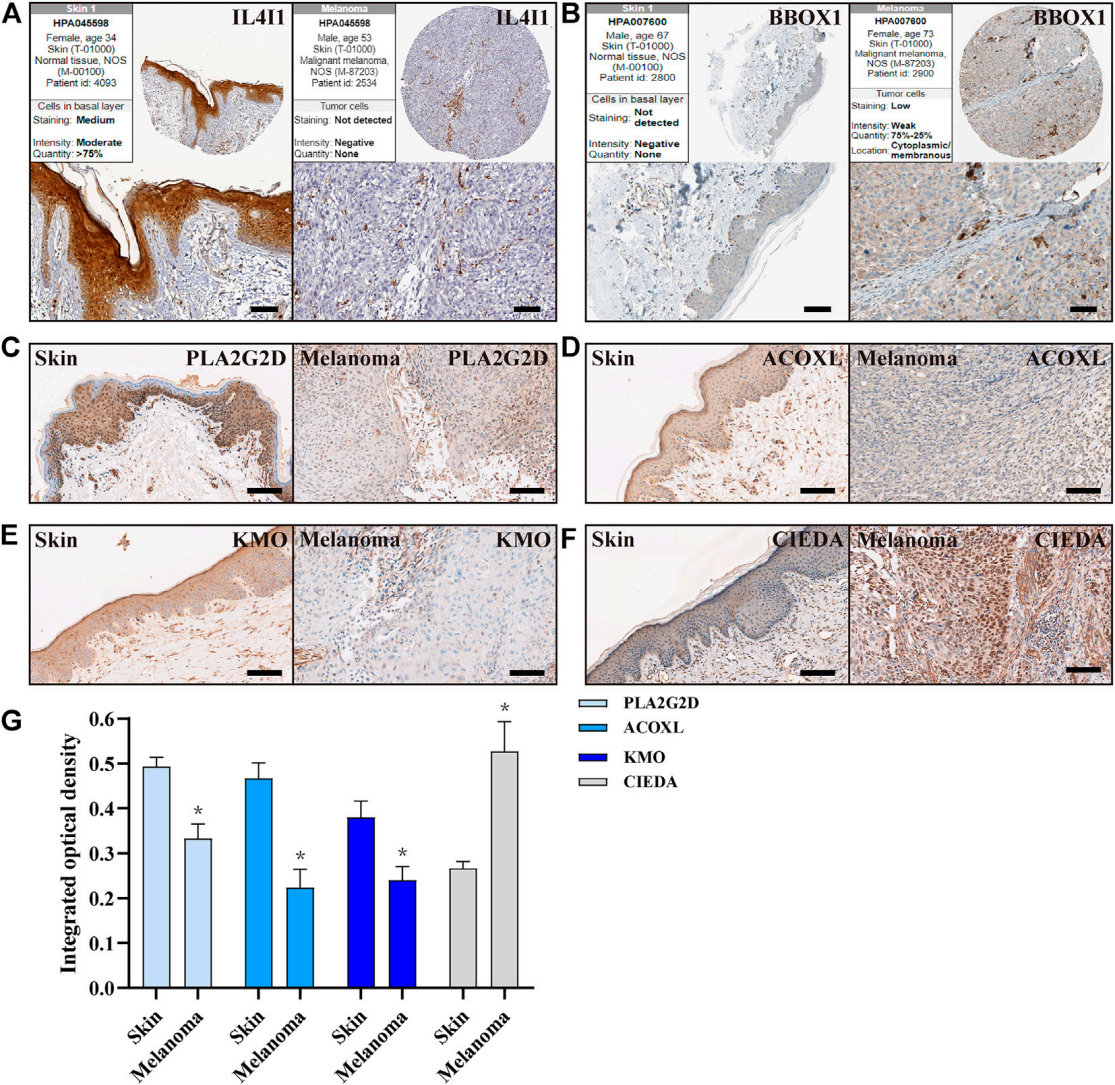
Expression of FA metabolism-related genes in normal skin and CM tissues. **(A,B)** IHC of the *IL4I1* and *BBOX1* in CM and normal skin tissues from HPA. **(C–F)** IHC of the *PLA2G2D*, *ACOXL*, *KMO,* and *CIDEA* in normal skin and CM tissues (n = 3), Scale bar = 100 µm. **(G)** Quantitative studies of *PLA2G2D*, *ACOXL*, *KMO,* and *CIDEA* were analyzed by counting the integrated optical density, respectively.

Finally, we further validated the expression of these key genes in normal skin and CM tissues. Melanoma arises from melanocytes in the epidermal layer of the skin, so we focused on the expression of these proteins in the epidermis. First, we searched the immunohistochemical slide information through the public database on the HPA website and found that the expression of *IL4I1* in normal skin epidermis was higher than that in CM tissue, while *BBOX1* protein was not detected in normal skin (Figures 8A,B). Next, our own immunohistochemical results showed that the expression of *PLA2G2D*, *ACOXL,* and *KMO* was higher in normal epidermal tissue than in CM tissue; however, the expression of *CIDEA* was reversed (Figures 8C–F).

## 4 Discussion

Currently, immunotherapy has been widely used in CM and achieved a good therapeutic effect in some CM patients. However, the therapeutic response rate of immunotherapies, particularly checkpoint inhibitors for CM patients remains low. It has been demonstrated that hot tumors usually respond to immunotherapies because they contain a lot of CD8^+^ T cell infiltrates with a higher immune score, high expression of immune checkpoints (e.g., *PD-1* and *CTLA4*), and active inflammatory response. On the other hand, cold tumors generally do not respond well to immunotherapy although switching cold tumors into hot tumors by promoting immune cell infiltration into the tumor environment is feasible (Herrera et al., 2021). Therefore, the discovery of biomarkers to distinguish between cold and hot tumors is particularly important for immunotherapy.

A recent study has reported that tumor cell metabolism is crucial for shaping the tumor microenvironment and its dysregulation is not only associated with the growth of tumors, but also with the therapeutic responses to immunotherapies (O'Sullivan et al., 2019). In addition, cancer cells can undergo metabolic reprogramming to support their survival when carcinogenic signals are blocked (DeBerardinis, 2020). FA, as a kind of important lipid molecule and energy source, is important for the growth of tumors and their therapeutic responses. The accumulation of FAs in the tumor microenvironment can affect the function and phenotype of immune cell infiltrates and FAO is crucial for CM metastasis and immune evasion (Li et al., 2018; Pascual et al., 2021). The accumulated FAs can limit anti-*CTLA-4* activity and inhibit tumor-specific and memory T cell infiltration into the tumors (Coutzac et al., 2020). In this study, we screened the FA metabolism-related DEGs in CM and found several DEGs had prognostic values in CM patients. Based on unique DEGs, we stratified CM patients into two clusters. In addition, we found that the frequency of CNV was critical for immune infiltrates in tumors and associated with the immunotherapeutic responses in CM patients, consistent with a previous report (Yang et al., 2021). Further analyses revealed that CM patients in Cluster 2 not only had more immune cell infiltrates (e.g., CD8^+^ T and B), higher immune infiltration score, and more active immune function (e.g., Inflammatory promotion) but also displayed a higher immune checkpoint activity, such as *CTLA4*, *CD274* (*PD-L1*), *HAVCR2* (*TIME3*), and *LAG3*. Hence, the CM patients in cluster 2 had most of the characteristics of a hot tumor and might respond better to immunotherapy.

It is well known that a high TMB is associated with better immunotherapeutic responses in tumor patients. Tumor with a high TMB usually has a higher level of neoantigens, which can promote immune cell infiltration, enhancing the effect of immunotherapy (Maleki Vareki, 2018). We found that the TMB in the cluster 2 CM was significantly higher than that of the cluster 1 and was expected to have better survival. Similarly, CM in cluster 2 displayed higher levels of *PD-1* and *CTLA4* expression and a lower IC_50_ for many potential immunotherapeutic agents. Therefore, cluster analysis of the FA metabolism-related genes in CM effectively stratified patients for rational immunotherapy.

To predict patients’ prognoses and explore the clinical application of FA metabolism-related genes in CM, we constructed a risk model using several FA metabolism-related DEGs, and validation revealed that this risk model had excellent sensitivity and specificity in separating CM patients for prognosis of OS in CM patients and their potential systemic therapy. Subsequently, we generated a nomogram using several independent risk factors, such as risk scores, age, tumor N stage, and T stage, and found that this nomogram had good validity and credibility for prognosis of CM patients.

Furthermore, five small-molecule drugs with potential therapeutic value were screened out through Cmap analysis, and they included ambroxol, tiletamine, mimosine, esculetin, and pizotifen. Previous studies have shown that tiletamine has potent cytotoxicity against melanoma cells by promoting ROS production and inducing cell cycle arrest, leading to melanoma cell apoptosis (Kyriakou et al., 2020). A combination of paclitaxel and ambroxol can synergistically kill lung cancer cells (He et al., 2020). Esculetin can inhibit the proliferation of pancreatic cancer cells to modulate their apoptosis by enhancing KEAP1 activity (Arora et al., 2016). Pizotifen has antitumor activity and is commonly used in gastrointestinal cancers. These small-molecule drugs may also have a potential therapeutic effect on CM (Jiang et al., 2020). Therefore, the FA metabolism can not only be used as a liquid biopsy method to quickly and effectively separate the cold from hot tumors to assist in rational immunotherapy but also predict patients’ prognosis and potential treatment drugs.

The DEGs for separating clusters in our study are associated with development and progression of several types of malignancies. *ALDH3A1* over-expression can enhance the secretion of *PD-L1* in melanoma cells *in vitro*, and the levels of *ALDH3A1* expression are consistently correlated with those of *PD-L1* and *COX-2* in clinical melanoma and lung cancer samples (Terzuoli et al., 2019). *ALOX12B*, an immunosuppressive factor, can inhibit immunity and promote tumor progression (Uderhardt et al., 2012; Rooney et al., 2015). *BBOX1* inhibitors can restrain the progression of triple-negative breast cancer (Liao et al., 2020). *CYP4F3* has been identified as a cancer promoter of lung cancer (Yin et al., 2017). *PLA2G2D* has been found to have potential as a potential biomarker of adaptive resistance to immune checkpoint inhibitors (Cindy Yang et al., 2021). However, *PLA2G2D* expression is associated with delayed tumor growth by enhancing anti-tumor immunity in a mouse model of skin cancer (Miki et al., 2013). The *IL-1β* expression is significantly upregulated in glioblastoma and negatively correlated with the expression levels of *KCNJ10*, suggesting that *KCNJ10* may promote inflammation in the TME (Brandalise et al., 2020). Except for *ALDH3A1*, the precise roles of other factors we studied in CM remain to be determined. Our findings may provide new insights into the mechanisms underlying the immune regulation of CM.

We recognized that our studies had limitations. First, we only obtained data from TCGA and other public databases, but we did not explore real clinical samples to validate our findings. Second, although several independent data sets were used for validation of our findings, the retrospective study in nature might have potential bias. Therefore, the reliability and values of this model need to be further validated by well-designed prospective, multi-center, large-scale studies. However, our findings may provide a reliable reference for the specific interpretation of metabolic reprogramming in CM. It is worth mentioning that the risk model and nomogram have good clinical prognostic value, and the cluster analysis may help improve the current predicament in predicting immunotherapeutic responses of CM patients. Furthermore, our cluster analyses and model may be valuable for development of new therapeutic strategies for precision medicine and personalized immunotherapy and may contribute to improving the prognoses of CM patients.

## 5 Conclusion

Our data indicated that the FA metabolism-related DEGs were effective for identification of hot tumors and improving immunotherapeutic responses and prognosis of CM. Exploring the mechanism underlying disordered FA metabolism may help not only for precision medicine but also for developing immunotherapy strategies for CM.

## Data Availability

The original contributions presented in the study are included in the article/Supplementary Material; further inquiries can be directed to the corresponding author.

## Supplementary material

Supplementary Figure S1 NMF of the external validation cohort and GSEA of clusters in the TCGA cohort. (A,B) All the heatmaps and NMF rank surveys of the external validation cohort. (C) GSEA of cluster 2 CM in the TCGA cohort. (D) GSEA of cluster 1 CM in the TCGA cohort.

Supplementary Figure S2 Prognosis value of the risk model.

Supplementary Figure S3 Clinical analyses of risk group CM patients. (A) t-SNE, PCA, and 3D PCA of risk groups. (B) GSEA of the low-risk group. (C) Immune heatmap of risk groups. (D) Correlation between risk scores and immune infiltrates. (E) Comparisons of immune-related scores in risk groups. **p*<0.05, ***p*<0.01, and ****p*<0.001. A *p* value of < 0.05 was considered to indicate a statistically significant difference.
