# Supplementary figures and images for: Transcriptome profiles of fatty acid metabolism-related genes and immune infiltrates identify hot tumors for immunotherapy in cutaneous melanoma
Source: Front Genet. 2022 Sep 19;13:860067. doi: 10.3389/fgene.2022.860067 (PMC9527329; doi:10.3389/fgene.2022.860067)

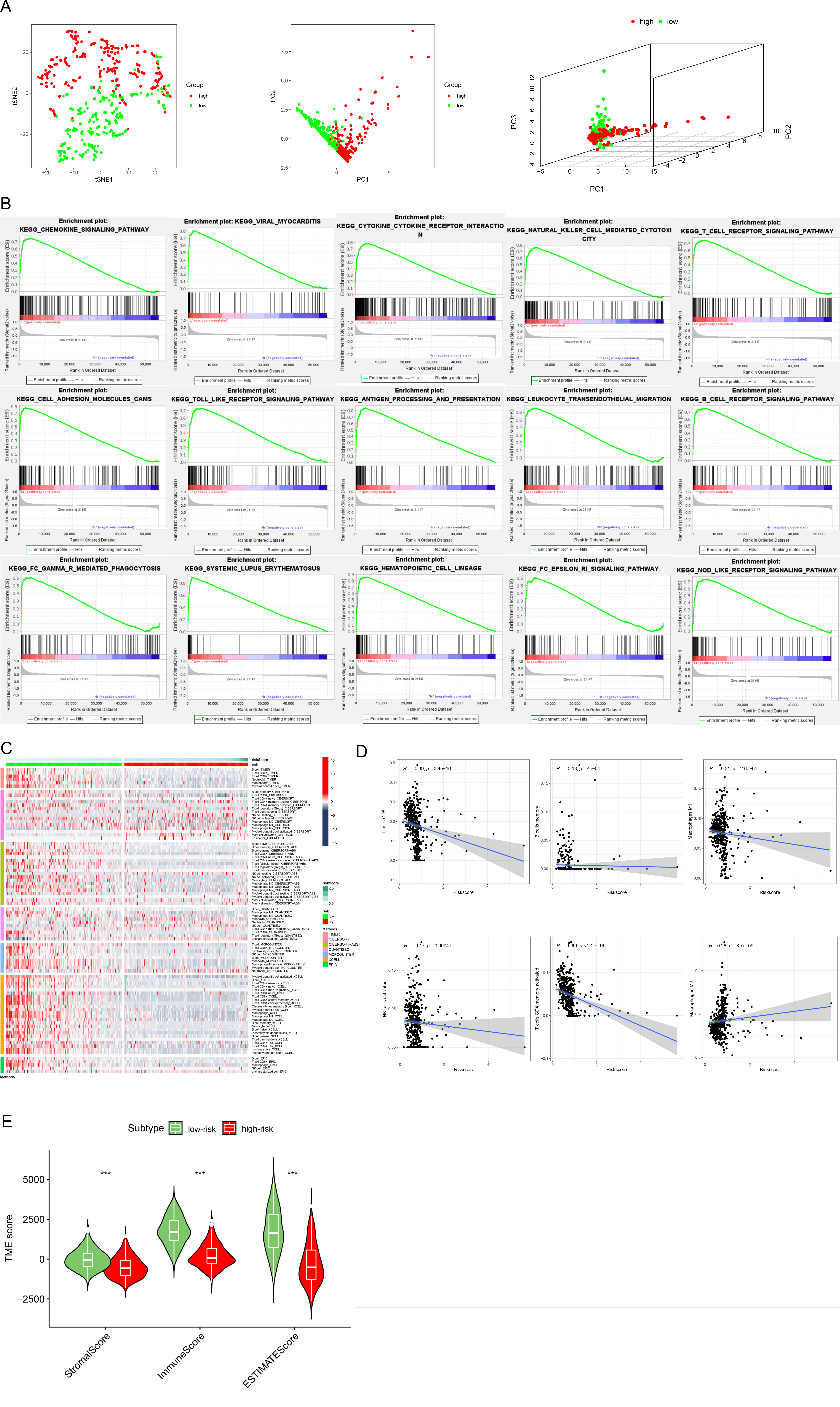

Supplement: Supplementary file 1 [file Image3.JPEG]

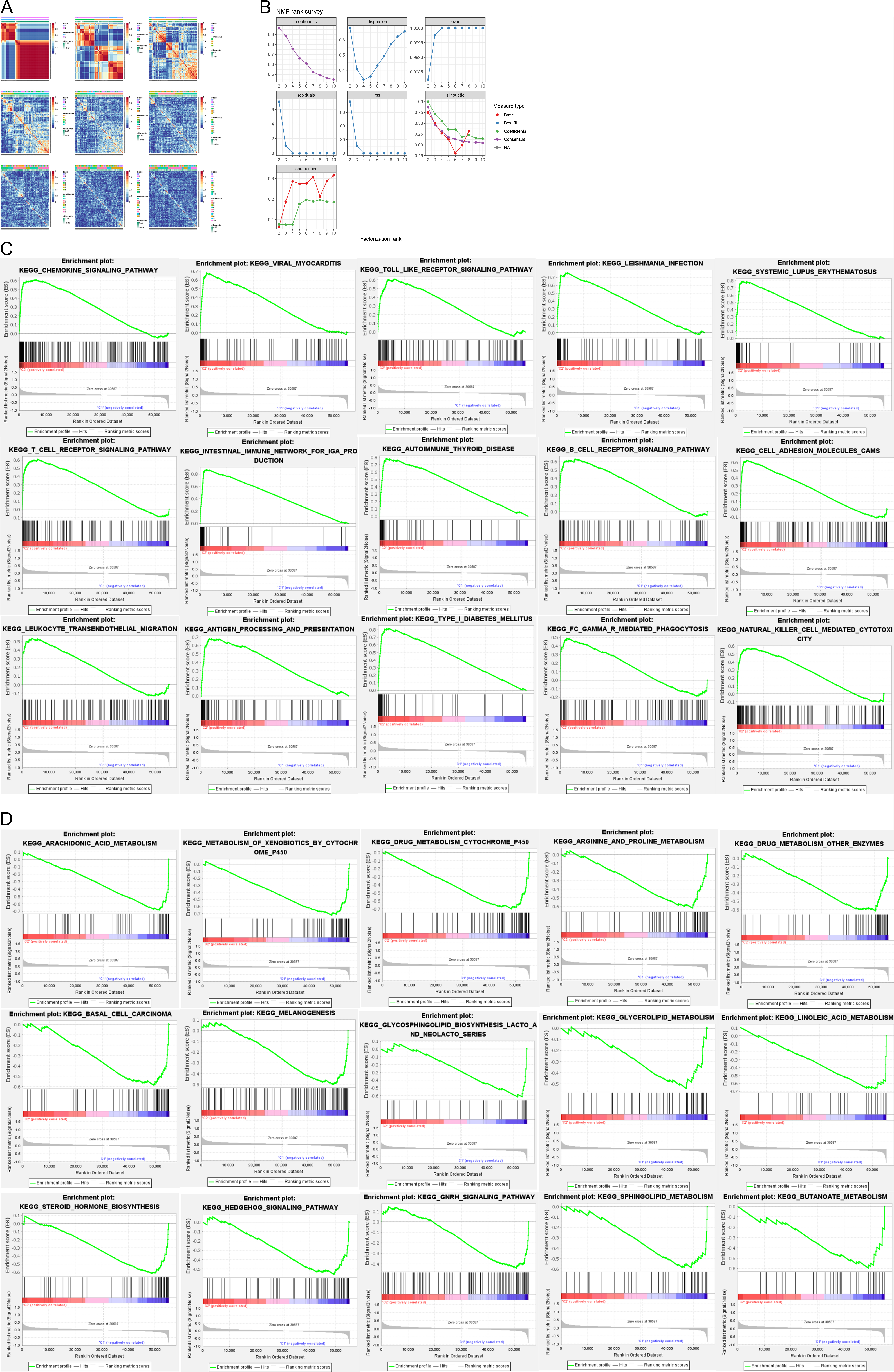

Supplement: Supplementary file 2 [file Image1.JPEG]

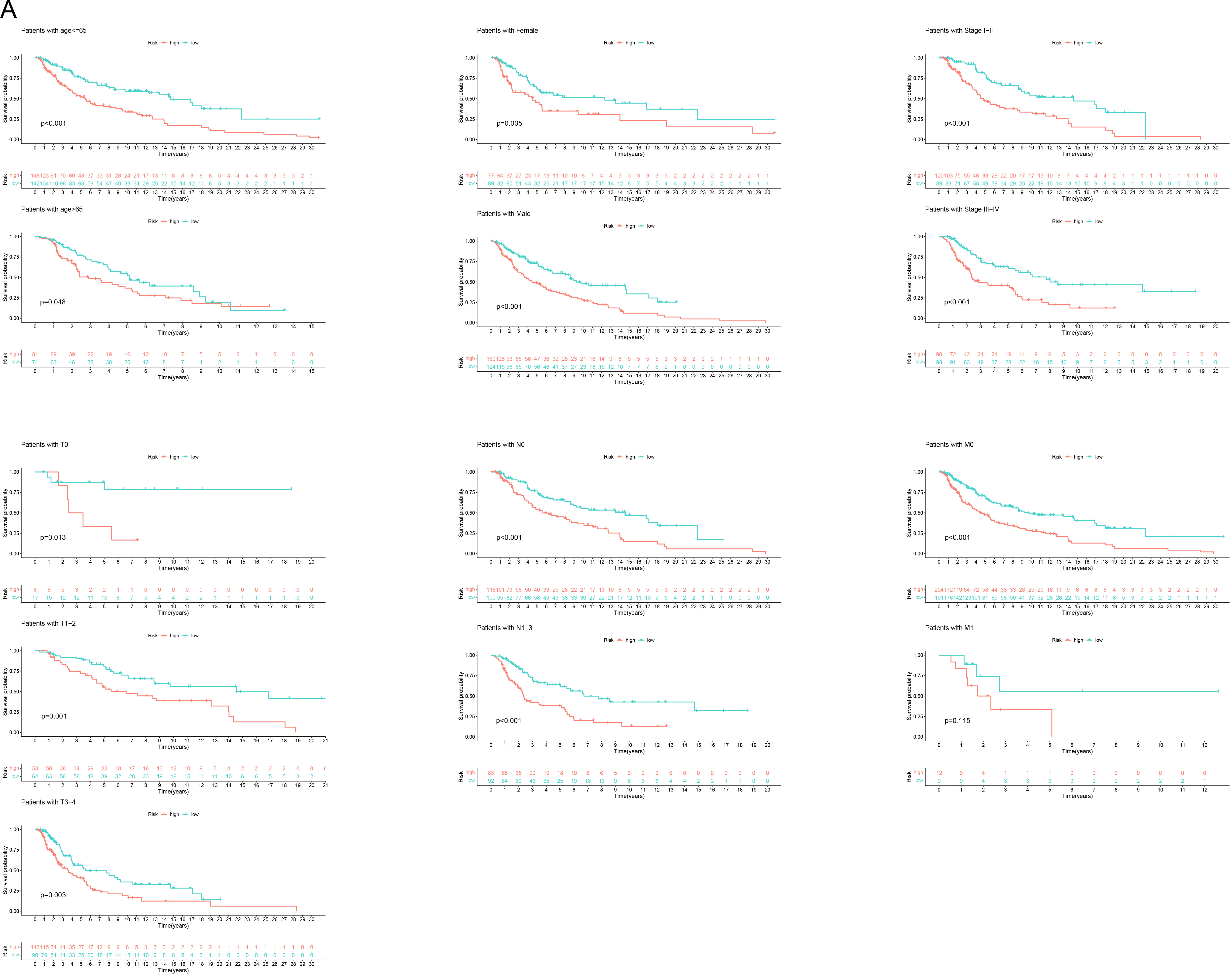

Supplement: Supplementary file 3 [file Image2.JPEG]
